# Supplementary material for: Same principle, but different computations in representing time and space
Source: Front Neurosci. 2024 May 7;18:1387641. doi: 10.3389/fnins.2024.1387641 (PMC11106375; doi:10.3389/fnins.2024.1387641)

Supplementary Material

# Supplementary Figures and Tables

## Supplementary Figures

**Figure S1.** Histogram of model comparisons. **(A)** Distribution of the difference between BLS2p and MLE2p models for time reproduction task **(B)** Distribution of the difference between BLS3p and MLE3p models for time perception. **(C)** Distribution of the difference between BLS3p and BLS2p models for time perception. **(D-F)** the same as **(A-C)**, but for distance reproduction task.

**Figure S2.** Subject No. 3 and observer model behavior in time reproduction (Up) and in distance reproduction (Down).

**Figure S3.** Subject No. 17 and observer model behavior in time reproduction (Up) and in distance reproduction (Down).
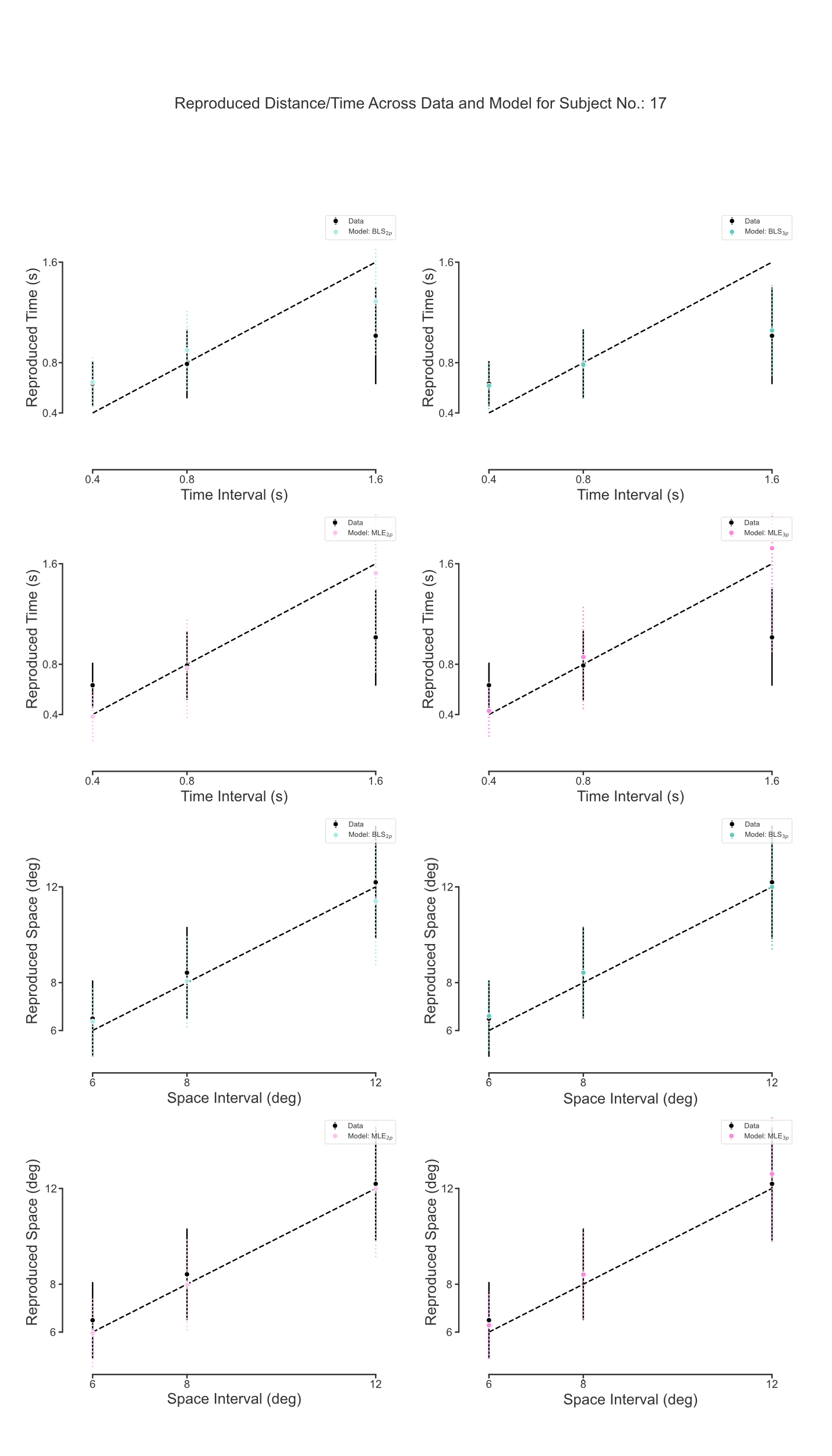

Supplement: Supplementary file 1 [file Data_Sheet_1.docx]
