# Supplementary figures and images for: Same principle, but different computations in representing time and space
Source: Front Neurosci. 2024 May 7;18:1387641. doi: 10.3389/fnins.2024.1387641 (PMC11106375; doi:10.3389/fnins.2024.1387641)

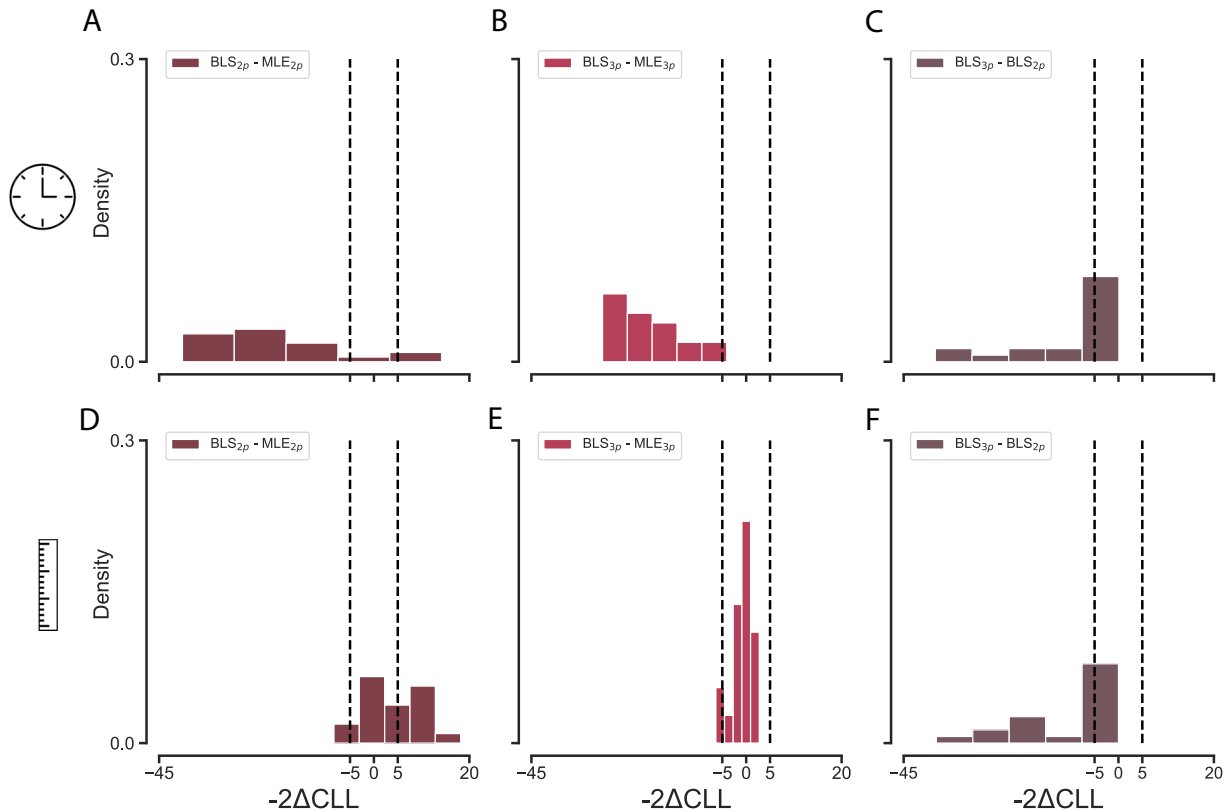

Supplement: Supplementary file 2 [file Image_1.pdf]

Reproduced Distance/Time Across Data and Model for Subject No.: 3

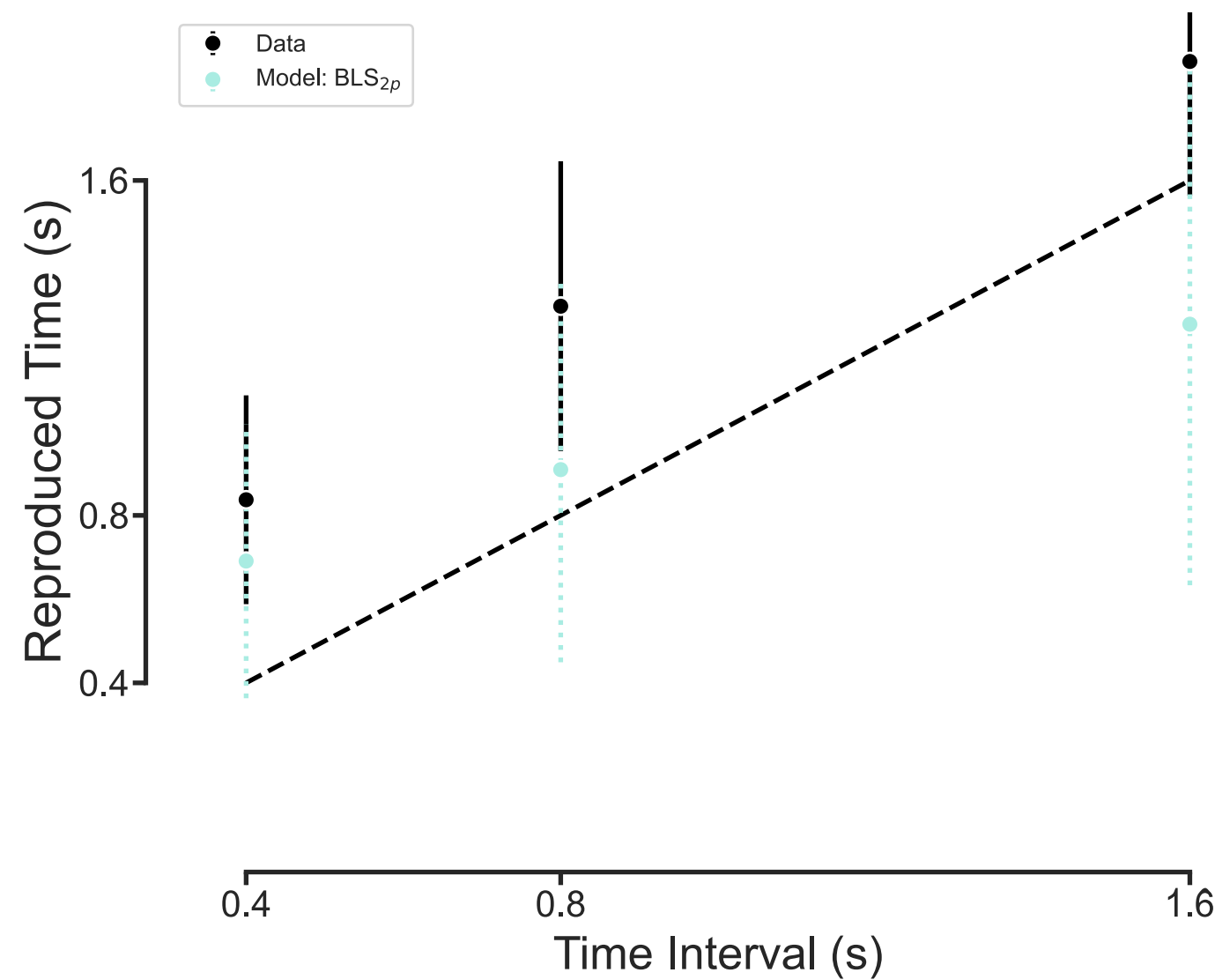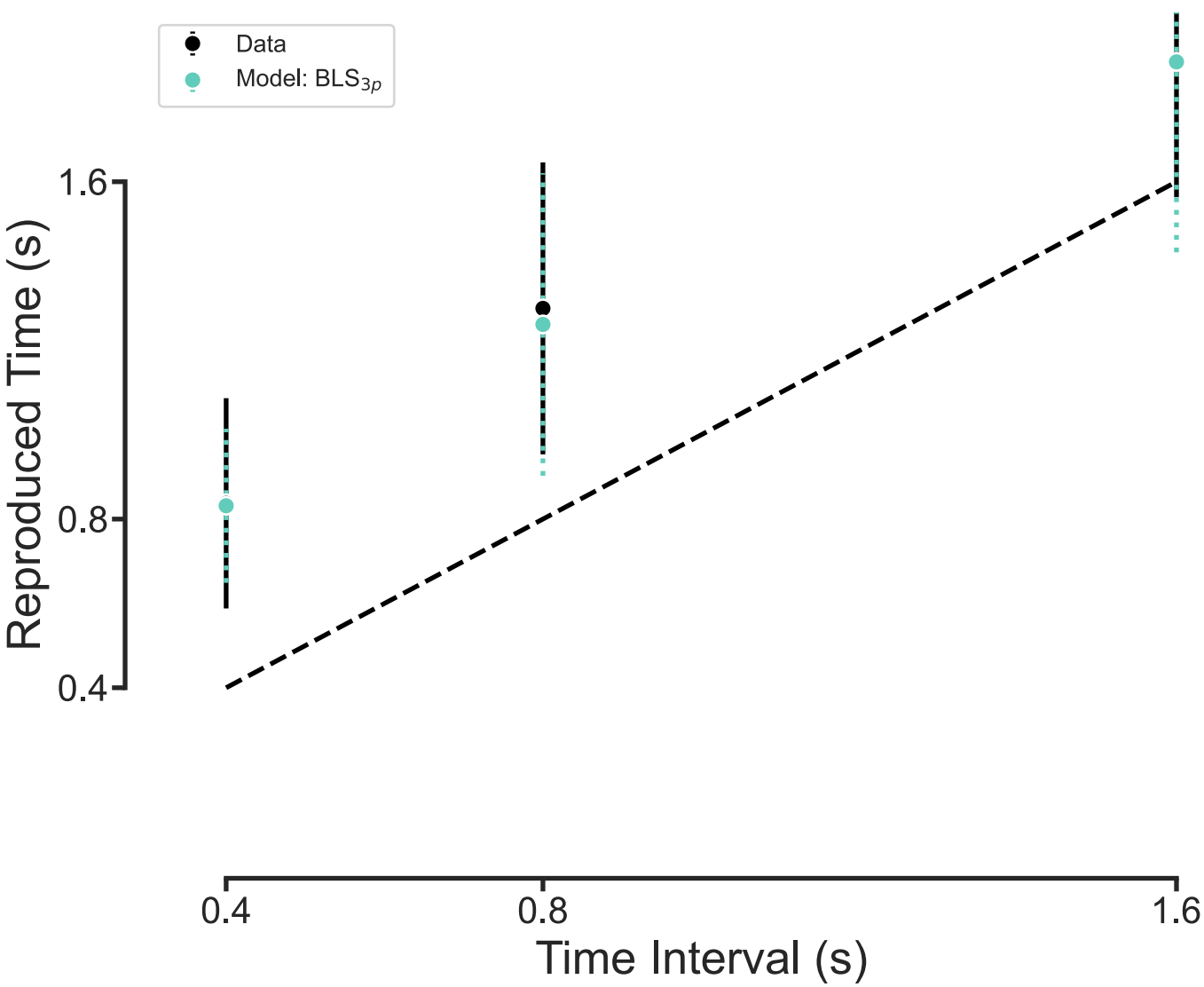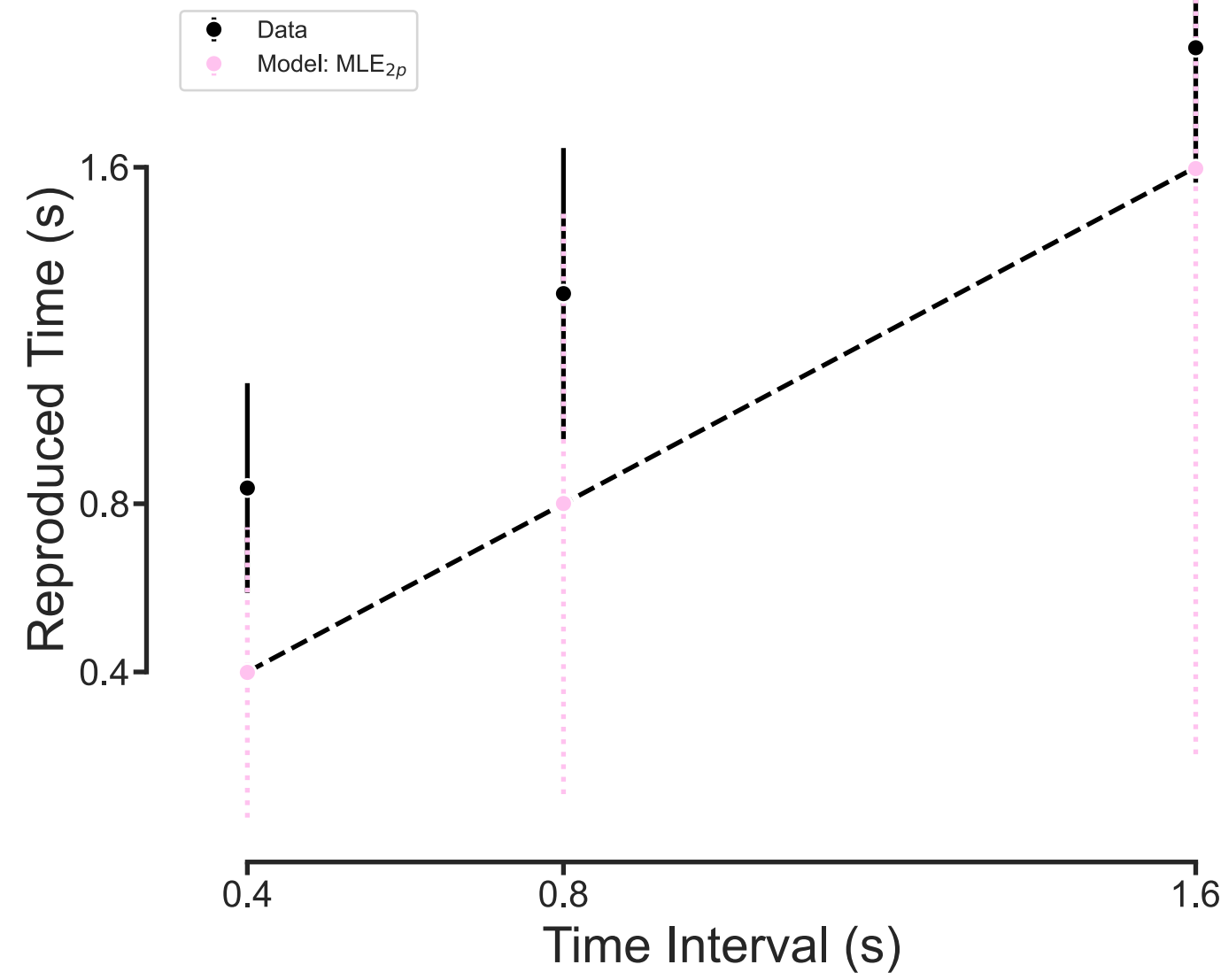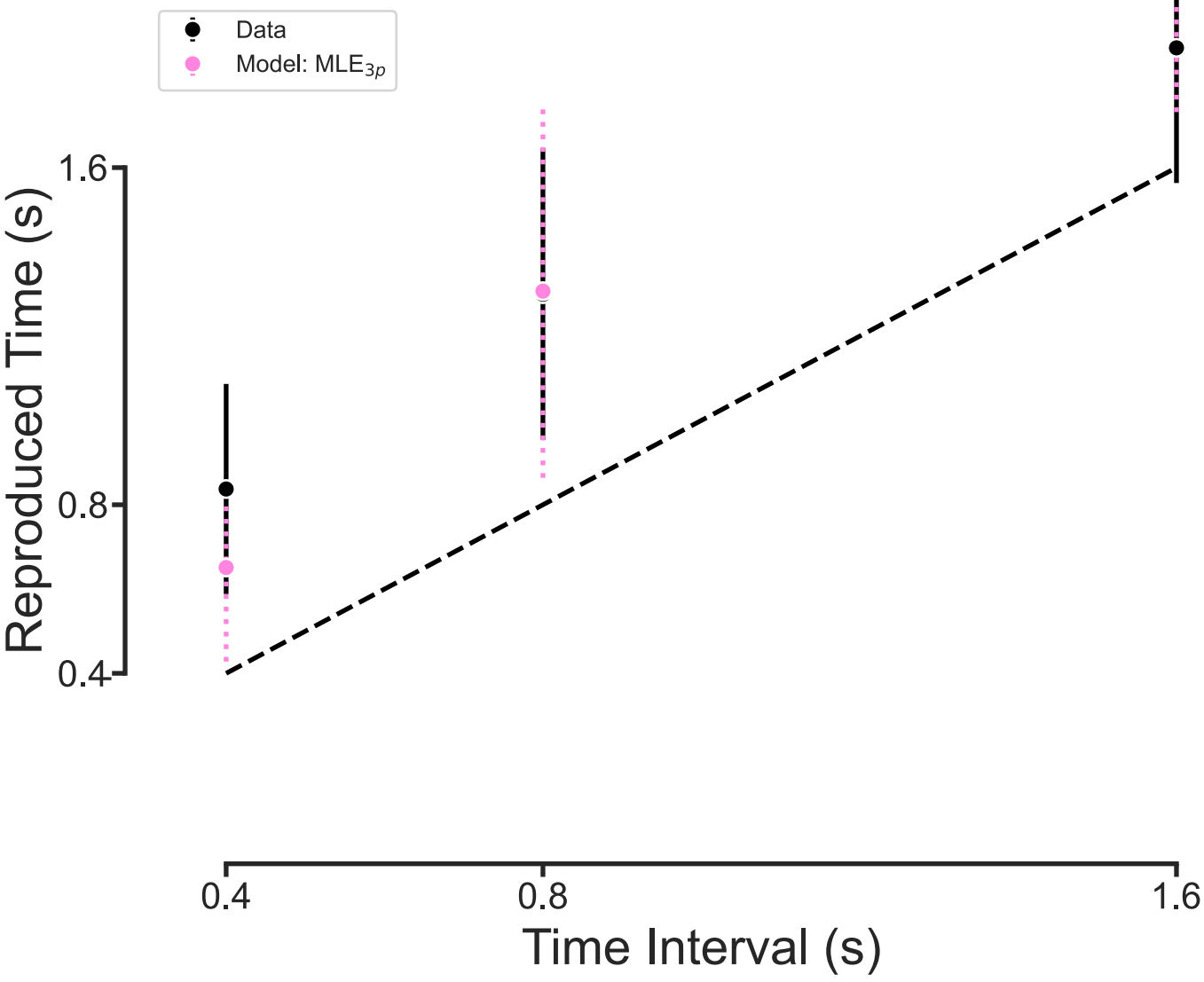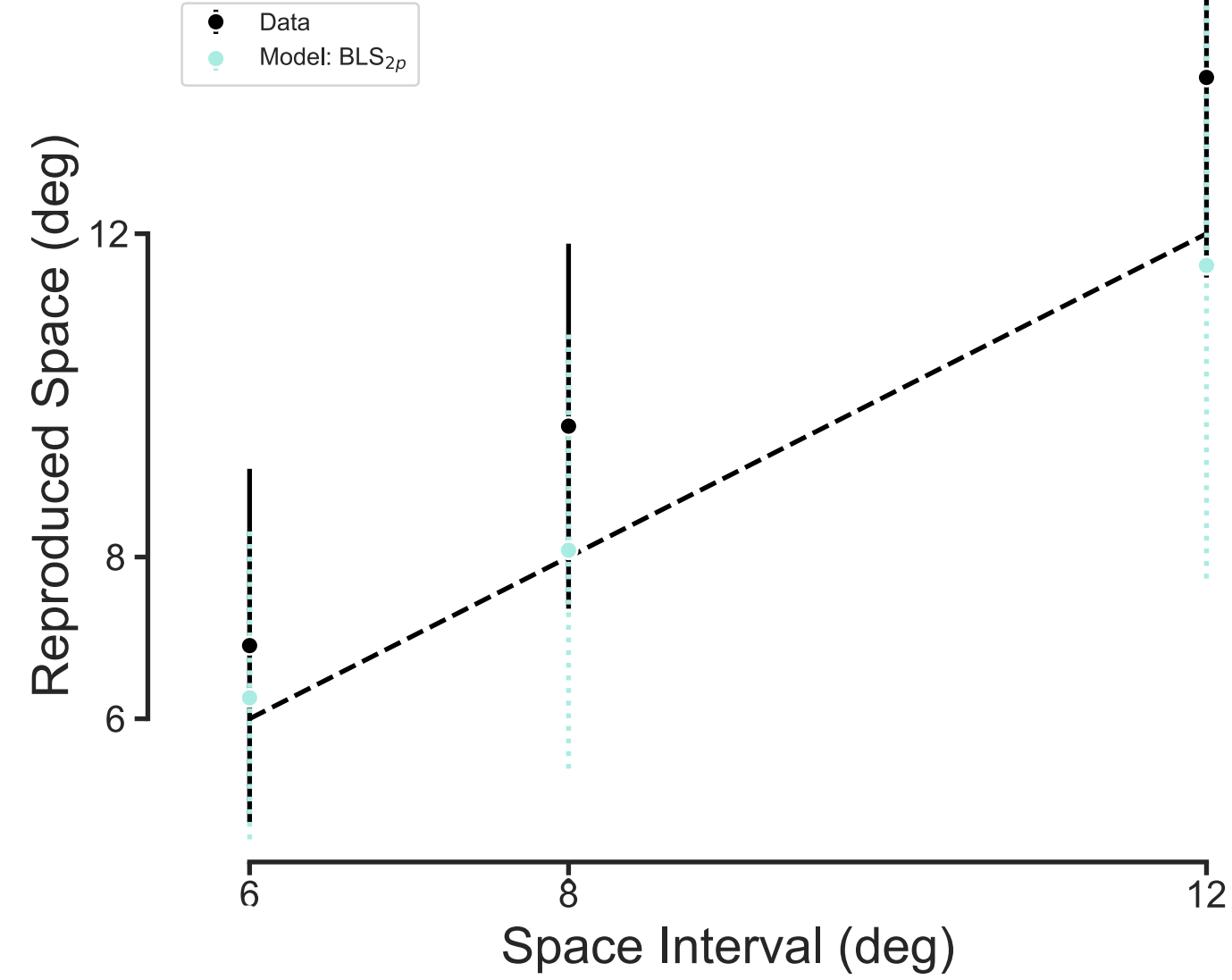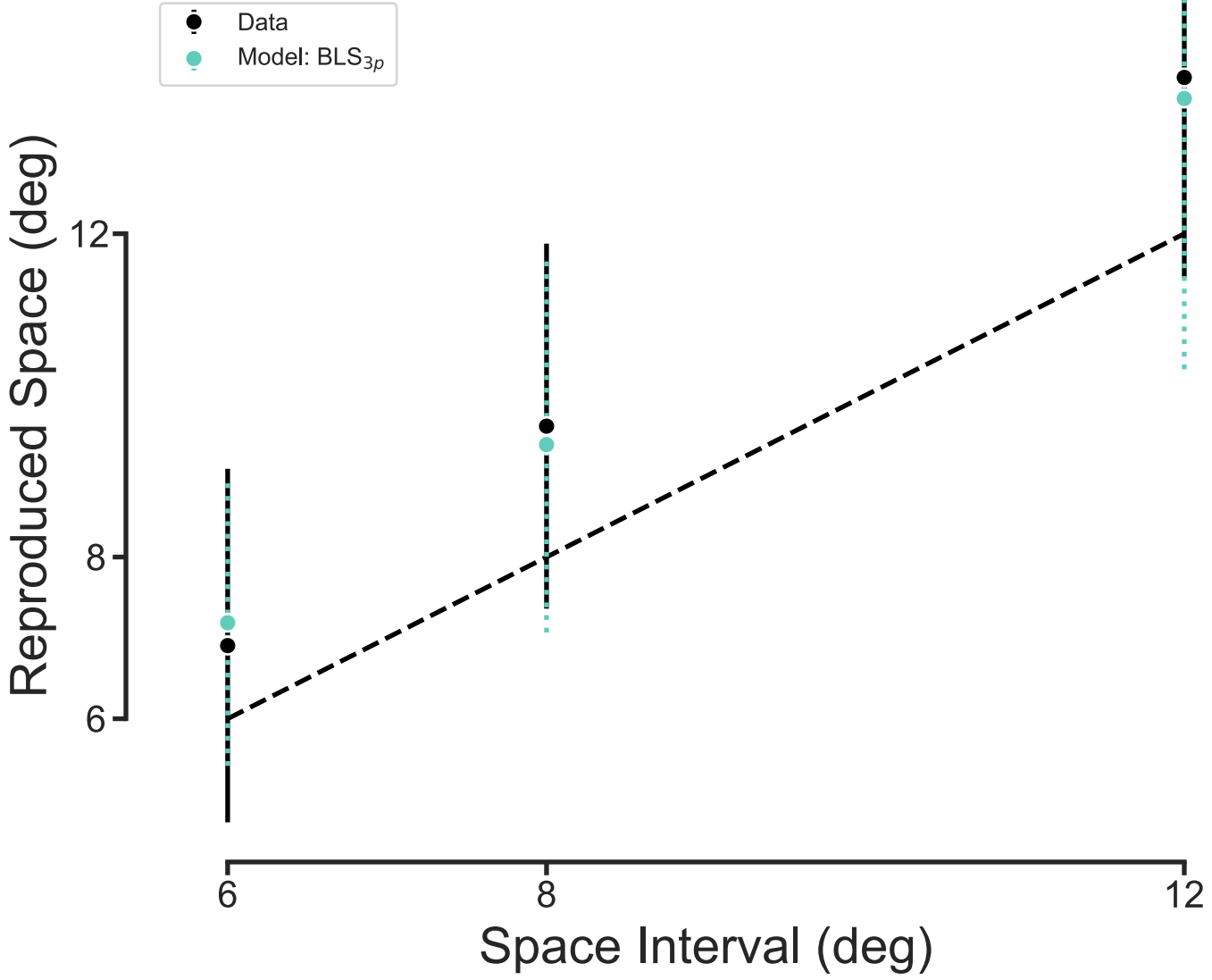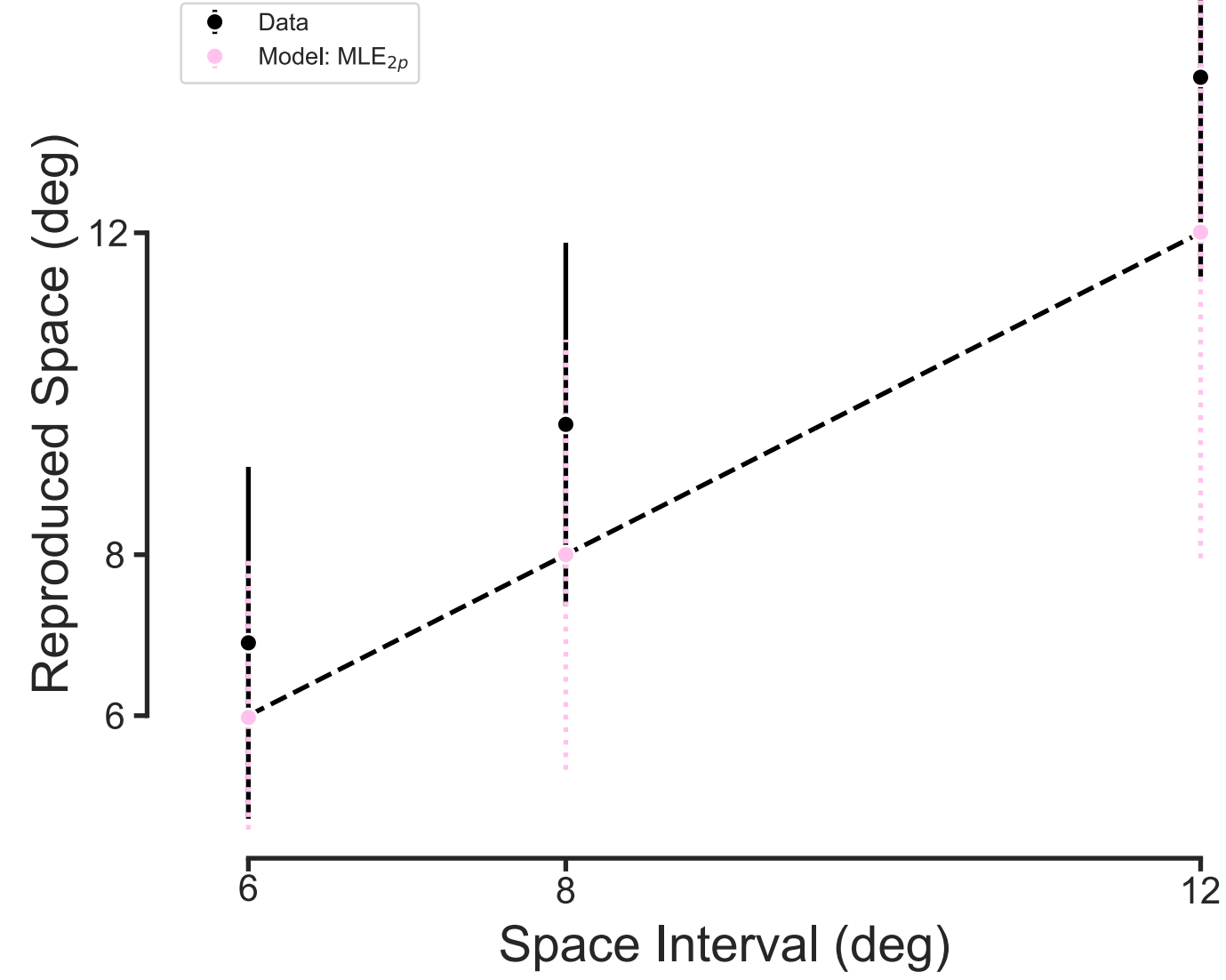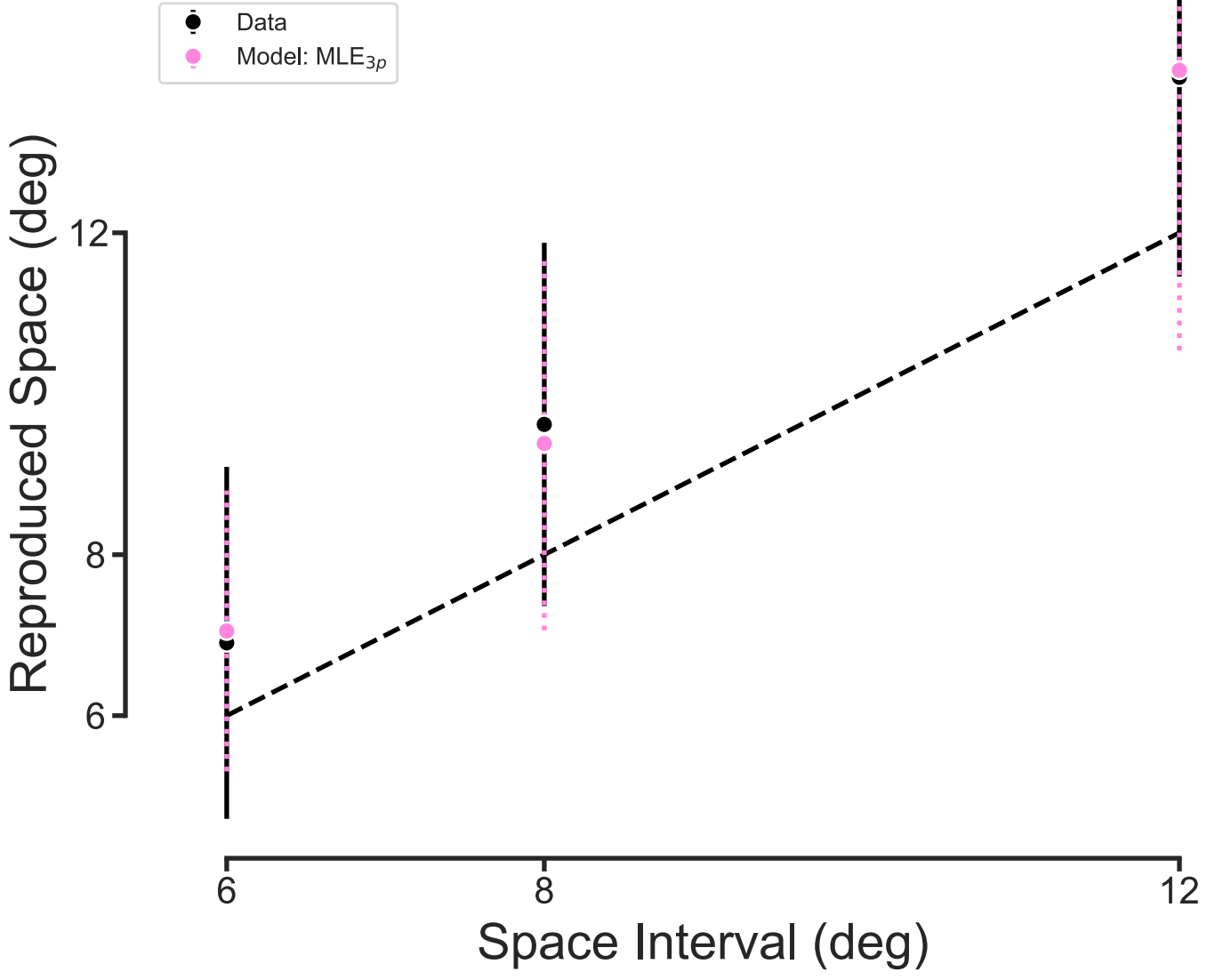

Supplement: Supplementary file 3 [file Image_2.pdf]

Reproduced Distance/Time Across Data and Model for Subject No.: 17

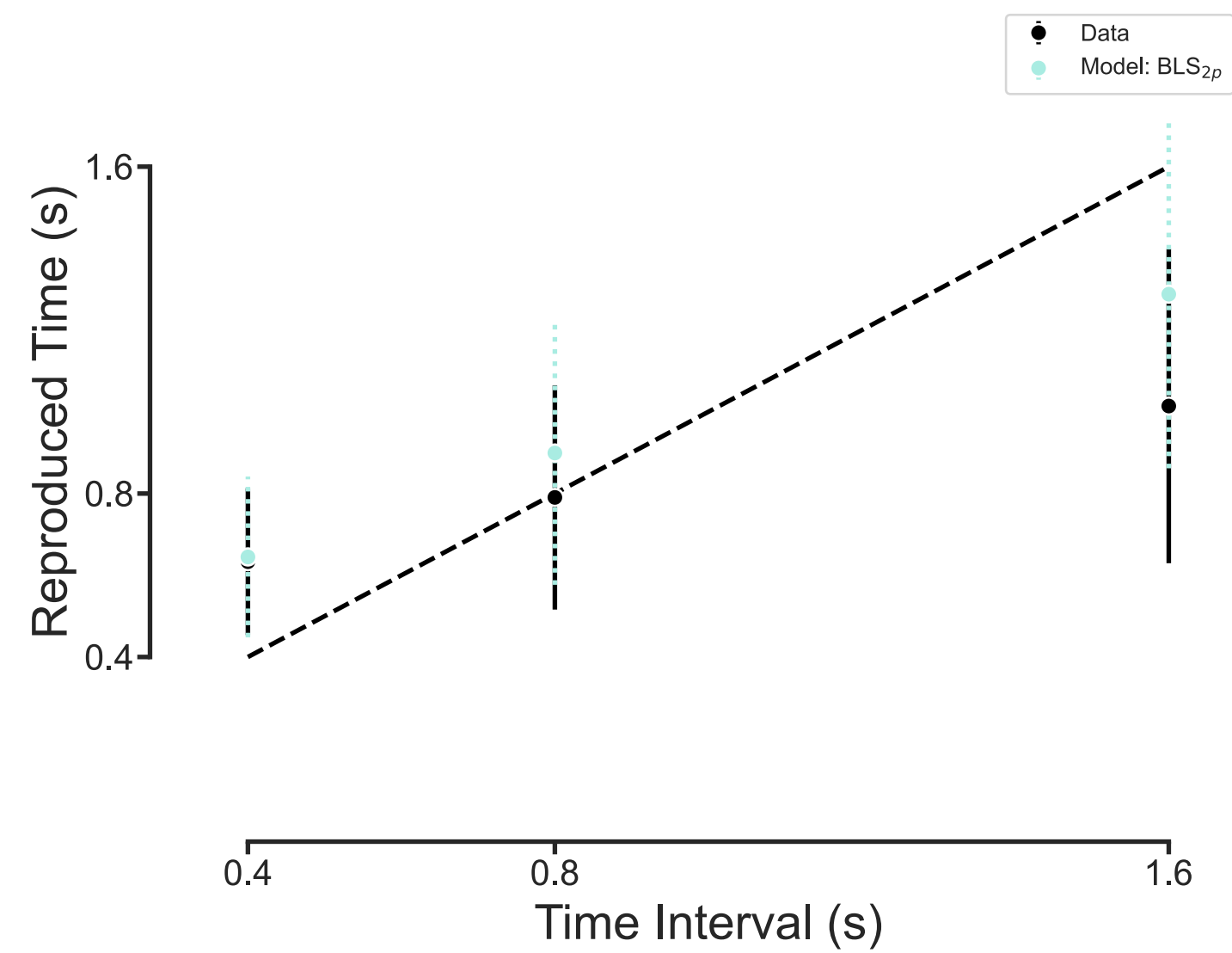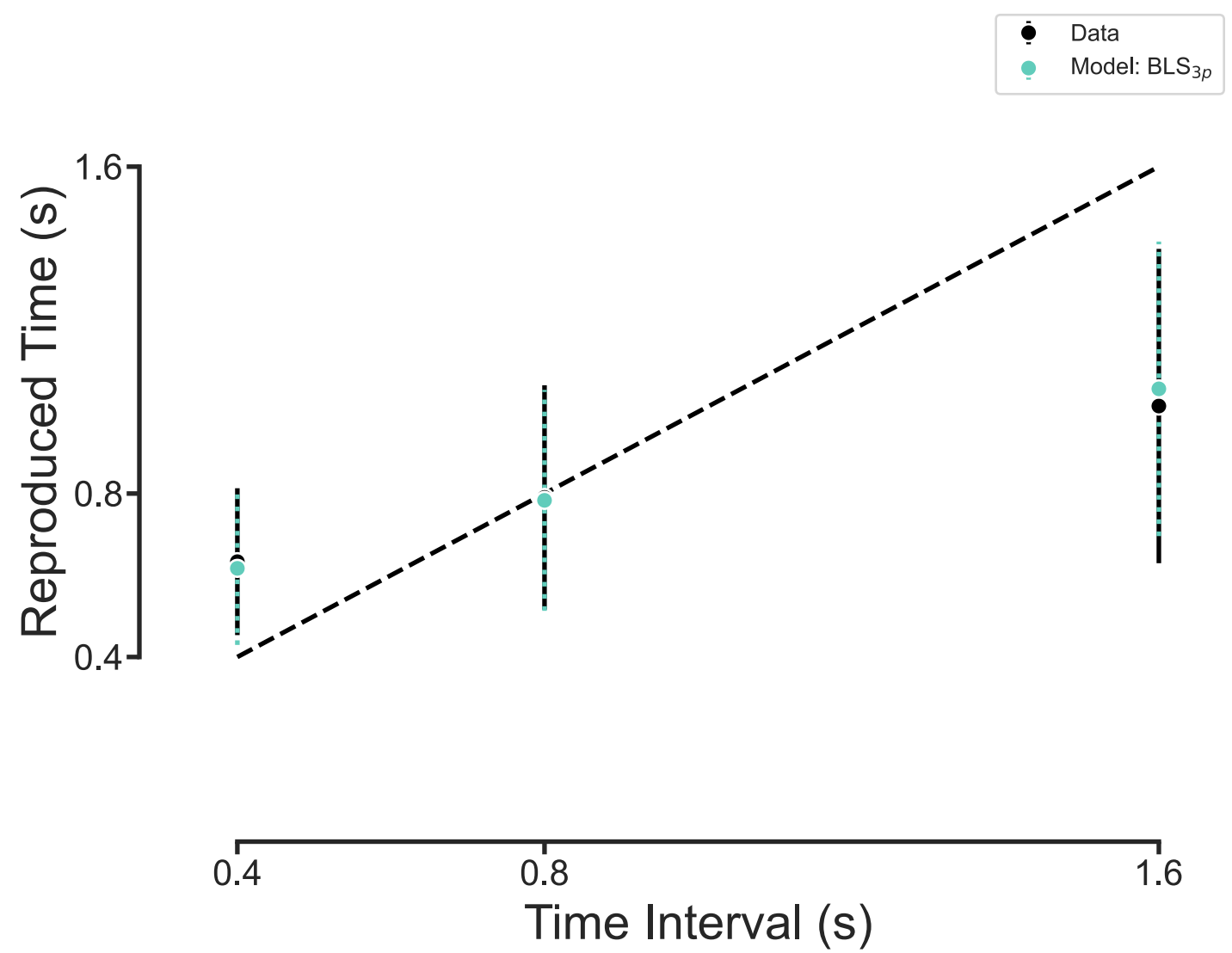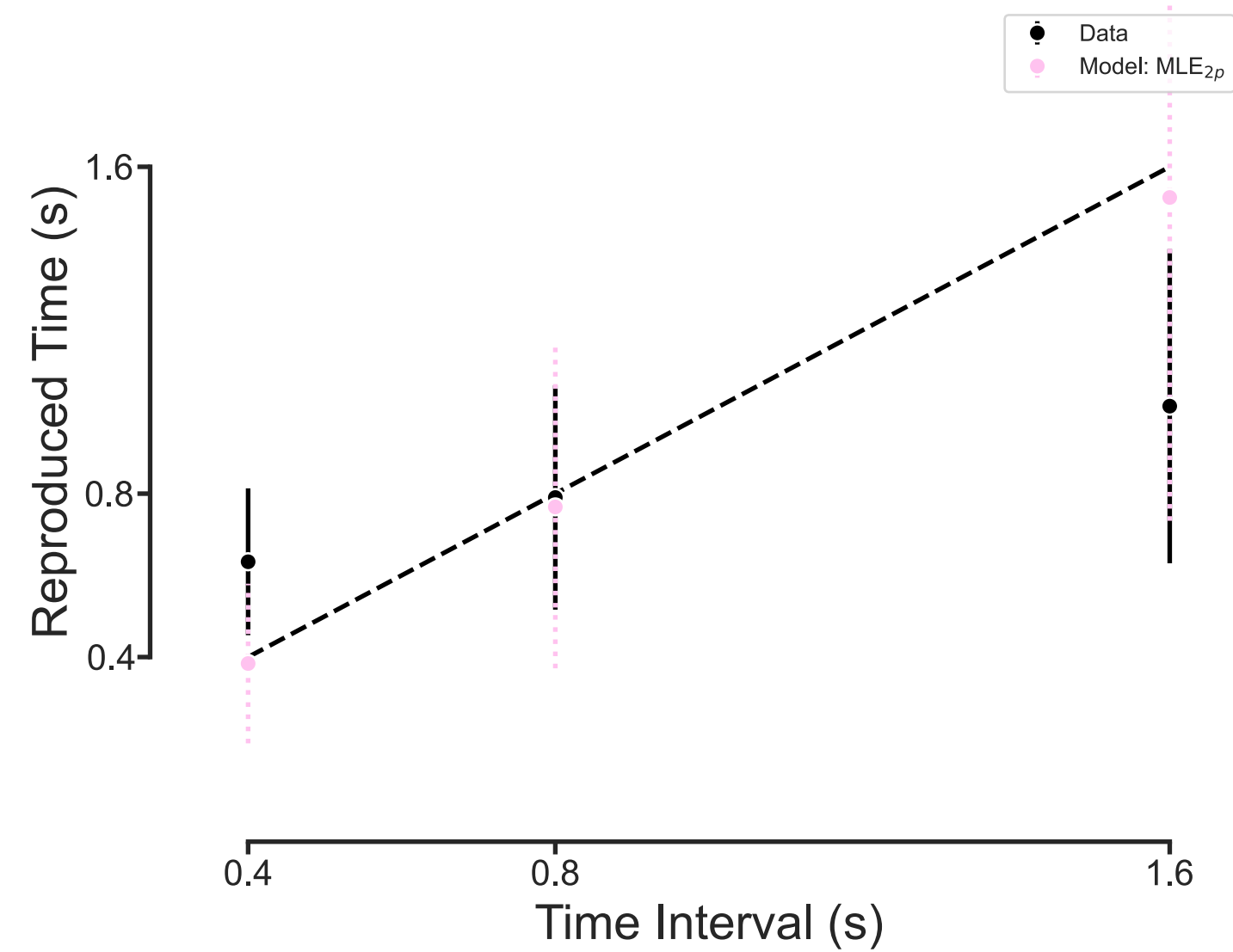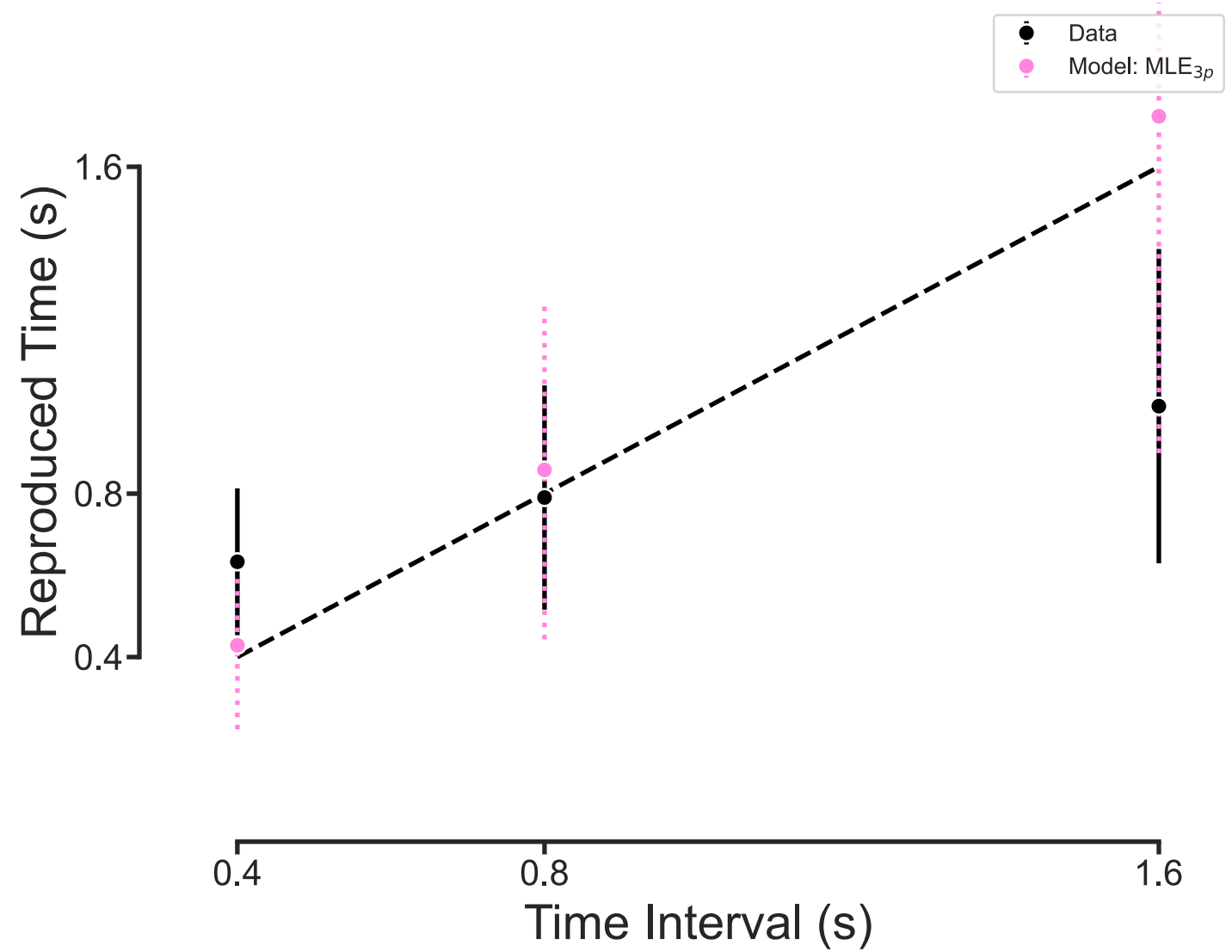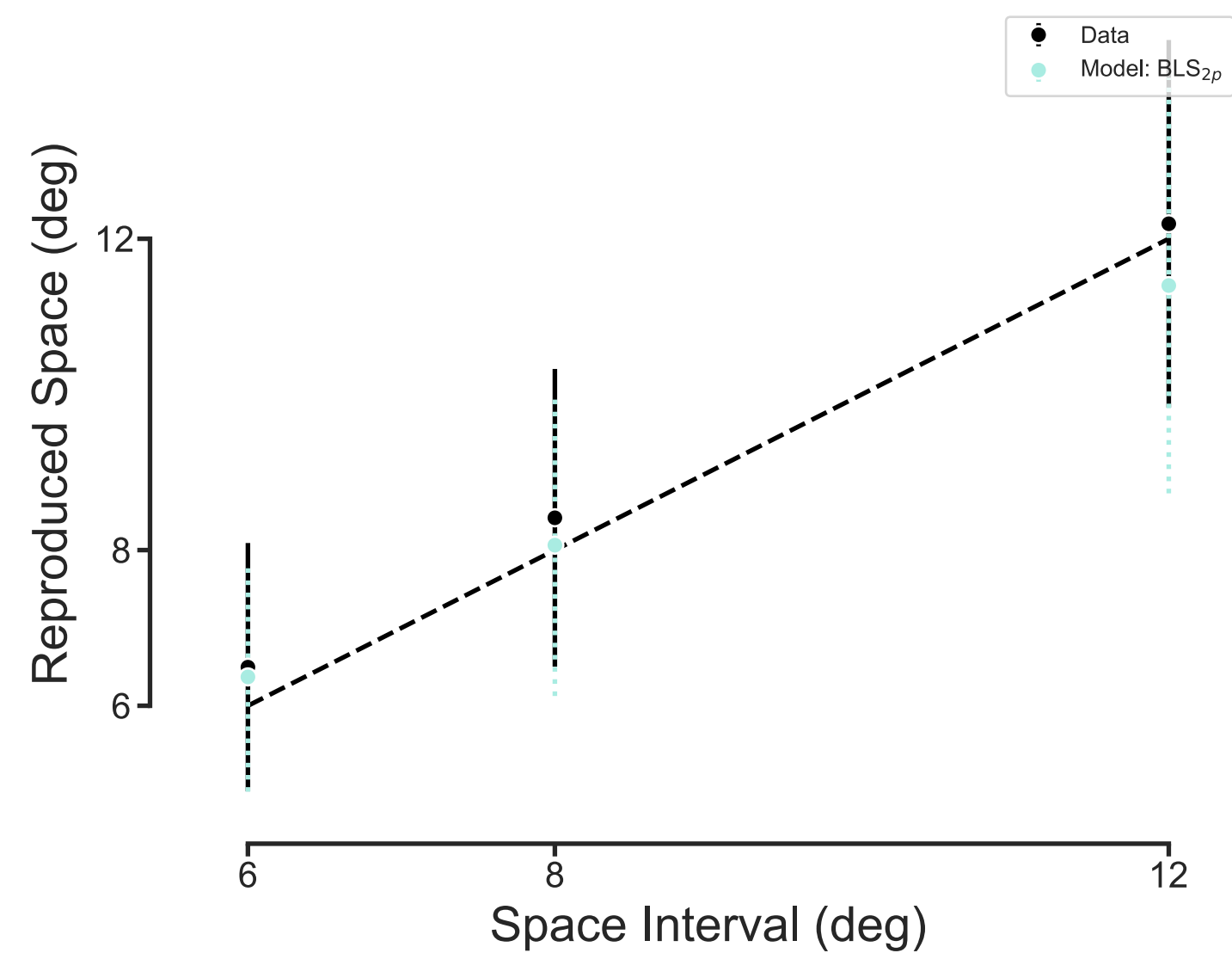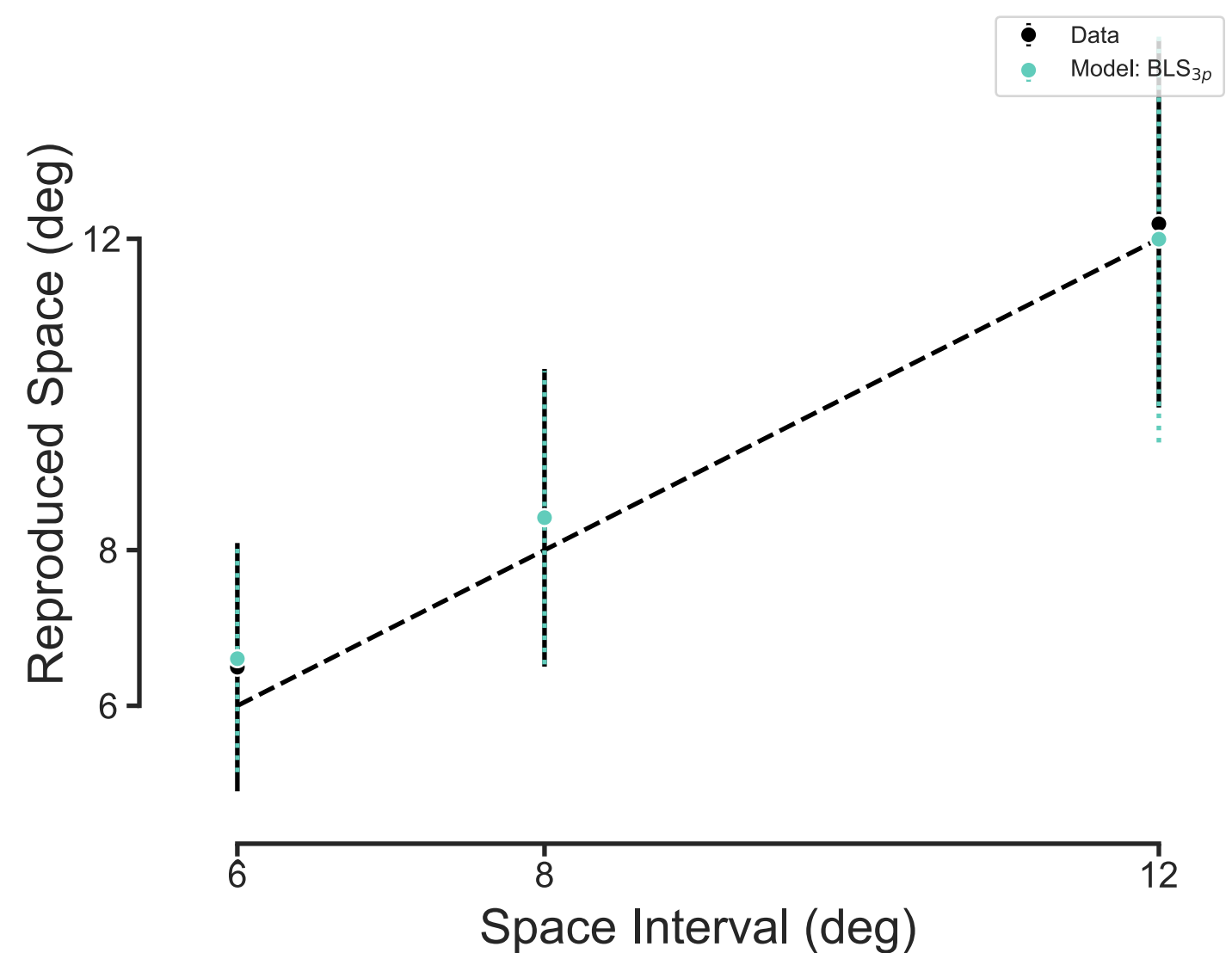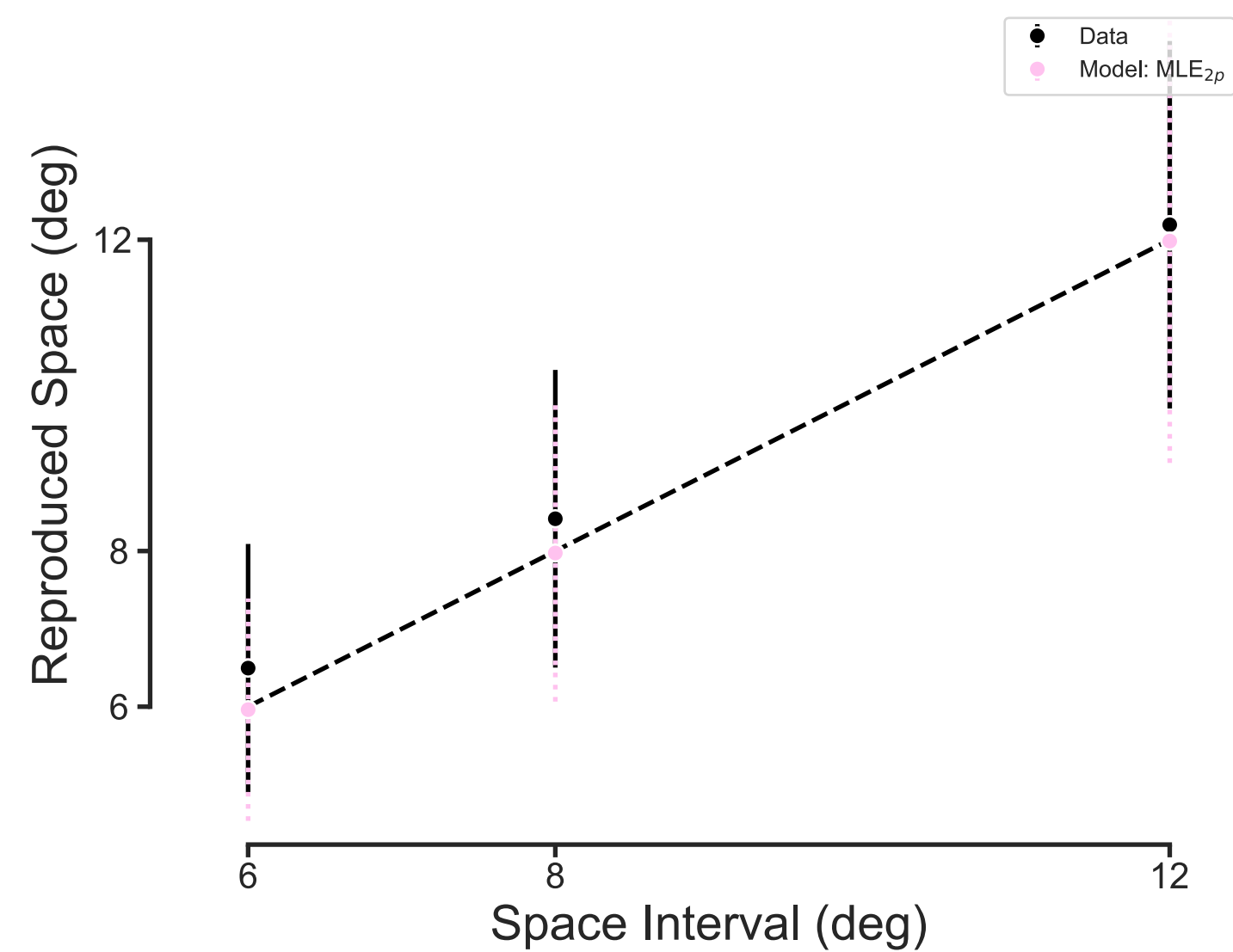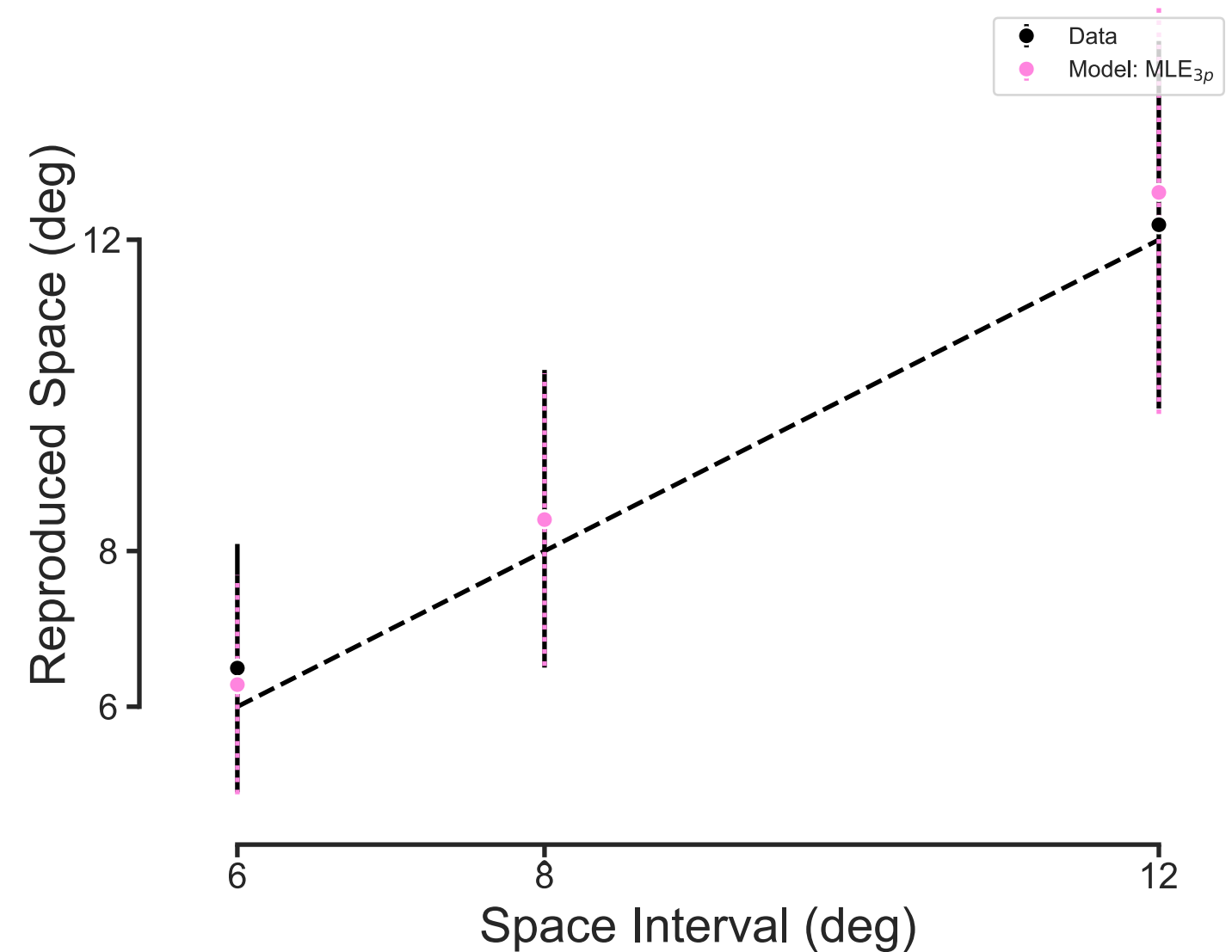

Supplement: Supplementary file 4 [file Image_3.pdf]
